# Migraine: translational challenges, novel therapies and disease modification

**DOI:** 10.1007/s00415-026-14231-z

**Published:** 2026-11-13

**Authors:** Leah Mistry, Alexandre Mathy, Anish Bahra

**Affiliations:** 1 Keble College, Parks Road, Oxford, OX1 3PG UK; 2 Neurosciences Department, John Radcliffe Hospital, Oxford University Hospitals, Headley Way, Oxford, OX3 9DU UK; 3 Department of Neurology, Great Western Hospital, Swindon, SN3 6BB UK; 4 Pain Management Centre @ National Hospital for Neurology and Neurosurgery, 25 Cleveland St, London, W1T3BF UK; 5 Neurosciences Department, John Radcliffe Hospital, Headley Way, Headington, Oxford, OX3 9DU UK

**Keywords:** Chronic migraine, Biopsychosocial, Disease modification, Pathophysiology, CGRP treatments, Chronic pain

## Abstract

Migraine is the most common neurological disorder, which after stroke confers the greatest neurological disability in the working population. As with other disorders of brain function, pathophysiological processes are more difficult to delineate, notwithstanding the biological complexities and clinical heterogeneity of the disorder. The current review will outline the framework upon which mechanistic pathways have been predicated. It will examine bench to bedside models: what we have learned from those which have been unsuccessful and address those which have borne fruit, presenting the clinical and exploratory insights gained. In the second part the more recent successes in drug therapeutics are summarised, from clinical trials to real world observations and limitations. With this in sight, the third part emphasises the need to reframe goals towards longer-term disease modification, addressing the impact of confounding comorbidities and moving towards a biopsychosocial model of pain.

## Introduction

Migraine is the most common disabling neurological disorder, conferring significant global disability [1]. In a third, the pain can be associated with aura, reversible neurological symptoms, typically visual, ± sensory/language symptoms; less commonly aura of assumed brainstem origin, largely audio-vestibular and rarely, hemiplegia [2]. The attack can be heralded by non-painful premonitory symptoms, most commonly fatigue, difficulty concentrating and neck stiffness [3]. Additional symptoms can include stimulus sensitivity, hunger and thirst, emotional and cognitive symptoms. The same symptoms inhabit the postdrome [4]. Understanding the anatomical substrate for the symptoms, the pathways and the neurotransmitters involved has provided the cornerstone to developing effective drugs. Yet a significant proportion remain drug-refractory. This update elaborates on the bench to beside journey and the remaining challenges. The first section addresses the basis for the anatomical and physiological substrates in head pain. The second section follows the chronological search for molecular targets within the pathways implicated, and evolution to therapies: those unsuccessful and the knowledge this brings, and efficacious therapies and their limitations. The final section will look at the management of the most refractory patients and reframe the goal towards disease modification.

### Anatomical infrastructure and neurotransmitter-targeted therapeutic drug development

Delineation of the fundamental physiological pain pathways was informed by early human neurostimulation and, anatomical mapping studies [5]. Penfield and McNaughton reported that in awake patients, stimulation of the superior sagittal sinus, a structure innervated primarily by the ophthalmic division of the trigeminal nerve (V1), caused pain in the brow, forehead, temple, occasionally the ear, neck and top of the head. Furthermore, stimulation produced 'nausea, yawning, restlessness and vomiting’ usually with pain, and abrupt fall of blood pressure which in one patient was confirmed simultaneously with precipitated severe pain and inability of the anaesthetist to ‘feel the patient’s pulse or read the blood pressure’. Furthermore, patients commented symptoms simulated those of their usual headache attacks [6].

Intracranial (IC) mapping of nociceptive fibres has been traced to the trigeminal nerve, primarily V1, nervus intermedius (sensory VII), glossopharyngeal (IX), vagus (X) nerves and cervical roots C2 and C3 [7]. Mapped alongside are autonomic fibres. The autonomic pathway is ascribed to connexions from hypothalamus, ultimately to peripheral predominantly parasympathetic, and sympathetic fibres. This in fact covers the breadth of distribution of symptoms observed in spontaneous attacks of migraine, such as neck stiffness, or ear pain. This often prompts initial referral to other departments, such as orthopaedics and physical therapy specialists, and otology for ear pain. The innervation is mainly around the arteries, veins and part of the dura, hence the original vascular hypothesis prompted by pain perception on experimental stimulation of the vasculature. The premonitory and prodromal symptoms, and the regulation of the vasculature, can be explained by the inherent autonomic connexions. It has become clearer that the vascular contribution to head pain may be epiphenomenal. There is dissociation in the pain and the vascular changes [8]. Sectioning of relevant fibres can remove the vascular phenomena without altering the expression of pain or response to treatment [9].

The nociceptive components of the V, VII, IX, X and upper cervical nerves run via respective ganglia to converge on second order neurons within the spinal trigeminal nucleus (STN), which extends from the brainstem to C3, hence becoming continuous with the dorsal spinal horn. This region incorporates the sensory information to connexions in the brainstem, forming not only structural, but functional connectivity observed in both animals [10] and humans [11]. It forms the rationale behind the effect of a greater occipital nerve block (GONB) on pain perceived within the distribution of the aforementioned nerves. Subsequently, the sensory fibres ascend in the contralateral spinothalamic tract to connexions in brainstem and diencephalon, namely hypothalamus, hence to thalamic nuclei and cerebral cortex.

Transmission of pain is controlled by the endogenous pain control system (EPCS) descending from cortex (prefrontal and sensorimotor) and brainstem, on to dorsal horn inhibitory interneurons; cranially, the spinal interneurons in the STN and dorsal horn of the upper cervical cord. The midbrain periaqueductal grey and the rostroventral medulla are key pain modulatory structures within the EPCS with opioid and serotonin receptors implicated [12, 13]. Eloquent functional imaging studies show that the expression of migraine pain coincides with alteration in interplay between the nociceptive and EPCS pathways [14].

About a third of migraine attacks are accompanied by aura [2]. Cortical spreading depression (CSD) is a neuronal and glial depolarization spreading across the cerebral cortex, posterior to anterior [15]. It has been proposed as a pathological correlate to aura and an initiating event to the migraine attack, following observation that the rate of progression of CSD is comparable to that of visual aura. Various hypotheses have been presented about how CSD can be implicated in precipitating the pain in both migraine with (MA) and without aura (MWA) [16].

Following observation of a familial tendency to migraine, genetic susceptibility was first confirmed in familial hemiplegic migraine (FHM), a rare autosomal dominantly inherited disorder. FHM1 is associated with gain-of-function mutations in CACNA1A, encoding the pore-forming alpha subunit of P/Q-type calcium channels (Ca_v_2.1), with around 25 pathogenic variants identified. FHM2 arises due to loss-of-function mutations in ATP1A2, which encodes the alpha-2 catalytic subunit of Na^+^–K^+^–ATPase, with over 80 causal variants. In FHM3, there are 11 identified gain-of-function mutations in SCN1A, encoding the voltage-gated Na^+^ (Na_v_1.1) channel [17]. Consistent with the putative correlation, in knockout mice with an FHM mutation, a much lower threshold for triggered CSD is observed [18]. Attempting to extrapolate from a monogenic disorder to more common MWA and MA, such mutations have not been found. However, variants in the KCNK18 gene, encoding a TWIK-related spinal cord potassium channel (TRESK), have been implicated in a family with migraine with typical aura [19]. Genome-wide association studies (GWAS) have identified several common susceptibility variants associated with migraine. Two recent large meta-analyses identified 123 and 79 risk polymorphisms, highlighting the highly polygenic nature of the disorder [20, 21].

### From bench to bedside

Glutamate can trigger CSD. It is released from structures integral to head pain pathways, binds to glutamate receptors in the STN and is increased in blood and cerebrospinal fluid during migraine attacks. A proof-of-concept study of the glutamate receptor antagonist BGG492 in acute treatment of migraine failed the set criterion compared to placebo [22].

More relevant, if considered a precursor to a migraine headache, a randomised controlled trial (RCT) of the CSD-inhibitor tonabersat was only effective in preventing attacks of MA but not MWA [23]. Of note, aura can occur before, during, after, without headache and, with all headache phenotypes, thus not only migraine, but other headache disorders, such as cluster headache, paroxysmal hemicrania, and hemicrania continua [24]. In addition, although observed in experimental animal and human studies, it has not clearly been shown to occur spontaneously in humans [25]. Therefore, although there may be an association of CSD with the aura phenomenon, there is no consistent association with the pain and occurrence in migraine without aura. In support of this, indirect pharmacological data have shown lamotrigine, an antiglutamatergic agent, has greater benefit in migraine with aura compared to migraine without aura [26].

Nitric oxide (NO) donors can trigger migraine attacks in predisposed humans. But, in sufferers of MWA and MA, only attacks of MWA are triggered. In FHM, there is no triggering effect, and pathophysiological processes do not seem to align with those in MA and MWA [27, 28]. In MA, there is an inherent increased risk of stroke not seen in MWA [29]. Comorbidity, thus, seems to best ascribe the association of aura with migraine.

The triggering ability has been used in animal models to elicit therapeutic targets within nociceptive pathways [30]. But selective inhibition of inducible NO synthase (GW274150) has not proven to be clinically effective as a preventative [31]. Although the NO animal model continues to be widely used, it is only relevant if it is known that the animals in question are predisposed to migraine and that a true attack has been triggered. Thus, although instructive in understanding physiology, use in pathophysiology is much more challenging.

## Novel treatments

### 5-HT_1F_ receptor agonists

The most successful bench-to-bedside translation has been the development of triptans, 5-HT (hydroxytryptamine)1B/D serotonin receptor agonists in acute attack treatment [32]. More recently, ditans, such as lasmiditan, were developed to avoid the vasoconstrictive effects associated with 5-HT1B/D receptor activation. The vasoconstrictive effects have been demonstrated in animal coronary arteries, but this has not been consistently observed in human studies and has not translated into increased cardiovascular risk in clinical populations [33–36].

The goal of acute treatment is pain relief within 2 hours, on the basis that around 70% of attacks last less than 4 hours [37]. Although superior to analgesics, as with all acute migraine treatments, triptans can cause exacerbation with frequent use, termed ‘medication-overuse headache’ (MOH). This phenomenon occurs faster with triptans than analgesics, and at lower frequencies and doses [38]. Crucially, preventative medications are less effective in MOH [39, 40]. Although the International Classification of Headache Disorders defines the critical frequency of overuse as 15 days/month for analgesics and 10 days for opioids and triptans, large population-based studies have shown that simple analgesics taken for > 8 days a month are associated with a 2–3% annual risk of progression from episodic (EM) to chronic migraine (CM), defined as headache on ≥ 15 days per month, of which ≥ 8 are migrainous, for at least 3 months [41]. A small Danish study showed individuals who abstained completely fared better than those restricting to 8 days per month [42]. At present, there are no data associating ditans with MOH, likely reflecting the short duration of available trials and lack of dedicated assessment of overuse risk [43].

The CM population accounts for the greatest proportion of global headache-related disability [1]. Individuals with prior treatment failures are often more resistant to subsequent therapies [44]. Therefore, preventative therapy remains central to successful management. Clinical trials aim to achieve a ≥ 50% reduction in mean monthly migraine days (MMD). This is based in part on a consistent placebo response of 30% [45] and recognition that higher response rates remain more elusive. However, it is becoming increasingly apparent that disability is not adequately represented by MMD. Most trials now use validated measures of function, using Patient-Reported Outcome Measures such as the Headache Impact Test (HIT-6) [46].

### CGRP-targeted treatments

Traditional preventatives have relied on repurposed medications, such as beta-blockers, anti-epileptics, and antidepressants [47]. Identification of calcitonin gene-related peptide (CGRP) released in humans during attacks of migraine led to the development of CGRP-targeted therapies, monoclonal antibody treatments (mAbs) and small-molecule receptor antagonists (gepants) [48] in acute and preventative treatment. Phase III RCTs have demonstrated efficacy of CGRP mAbs in the prevention of EM and CM, with favourable tolerability [49]. Across pivotal studies, 40–60% of individuals achieved a ≥ 50% reduction in MMD after three months of treatment (Table 1, 2, 3, 4).

**Table 1:** Pivotal clinical trials of CGRP monoclonal antibodies for the prophylaxis of episodic migraine

Drug	Key clinical trial	*N*	Retention (%)	Primary endpoint (≥ 50% responder rate) OR vs placebo (95% CI) where reported
**Erenumab** CGRP receptor Human monoclonal antibody (SC) 4 weekly	**STRIVE** (2017) [126] 6 months Prior preventative use: 42% Baseline MMD: 8.3/month	**Placebo (316)** ** 70 mg (312)** ** 140 mg (318)**	89.8%	**26.6%** **43.3%** 2.13 (1.52 to 2.98) **50.0%** 2.81 (2.01 to 3.94)
**Galcanezumab** CGRP ligand Humanised monoclonal antibody (SC) 4 weekly	**EVOLVE-1** (2018) [127] 6 months Prior preventative use: 60% Baseline MMD: 9.1/month	**Placebo (425)** ** 120 mg (210)** **240mg (208)**	81.9%	**38.6%** **62.3%** 2.6 (2.0 to 3.4) **60.9%** 2.5 (1.9 to 3.2)
**Fremanezumab** CGRP ligand Humanised monoclonal antibody (SC) 4 weekly	**HALO EM** (2018) [128] 3 months Prior preventative use: 61% Baseline MMD: 9.1/month	**Placebo (290)** **Single Higher Dose** **(288)** **Monthly (287)**	90.4%	**27.9%** **47.7%** 19.8 (12.0 to 27.6) **44.4%** 16.5 (8.9 to 24.1)
**Eptinezumab** CGRP ligand Humanised monoclonal antibody (IV) 3 monthly	**PROMISE-1** (2020) [129] 3 months Prior preventative use: NR Baseline MMD: 8.6/month	**Placebo (222)** ** 30 mg (223)** ** 100 mg (221)** ** 300 mg (222)**	94.0%	**37.4%** **50.2%** 1.7 (1.2 to 2.5) **49.8%** 1.7 (1.1 to 2.4) **56.3%** 2.2 (1.5 to 3.2)

Episodic migraine (EM) is defined as < 15 monthly migraine days (MMD). Retention refers to participants completing the double-blind treatment phase. Efficacy is defined as the proportion of patients achieving a ≥ 50% reduction in MMD at the primary efficacy endpoint of each trial. Odds ratios (ORs) are reported relative to placebo with 95% confidence intervals (CI). *NR* not reported, *SC* subcutaneous injection, *IV* intravenous administration

**Table 2:** Pivotal clinical trials of CGRP monoclonal antibodies for the prophylaxis of chronic migraine

Drug	Key clinical trial	*N*	Retention (%)	Primary endpoint (≥50% responder rate) OR vs placebo (95% CI) where reported
**Erenumab** CGRP receptor Human monoclonal antibody (SC)	**Tepper et al.,** (2017)[130] 3 months Prior preventative use: 68% Baseline MMD: 18.0/month	**Placebo (281)** ** 70 mg (188)** ** 140 mg (187)**	97%	**23.0%** **40.0%** 2.2 (1.5 to 3.3) **41.0%** 2.3 (1.6 to 3.5)
**Galcanezumab** CGRP ligand Humanised monoclonal antibody (SC)	**REGAIN** (2018)[131] 3 months Prior preventative use: 78% Baseline MMD: 19.4/month	**Placebo (538)** ** 120 mg (273)** ** 240 mg (274)**	93.1%	**15.4%** **27.6%** 2.1 (1.6 to 2.8) **27.5%** 2.1 (1.6 to 2.8)
**Fremanezumab** CGRP ligand Humanised monoclonal antibody (SC)	**HALO CM** (2018) [132] 3 months Prior preventative use: 21% Baseline MMD: 16.2/month	**Placebo (371)** **Quarterly (375)** **Monthly (375)**	91.5%	**18.0%** **38.0%** **41.0%**
**Eptinezumab** CGRP ligand Humanised monoclonal antibody (IV)	**PROMISE-2** (2020)[133] 3 months Prior preventative use: NR Baseline MMD: 16.1/month	**Placebo (366)** ** 100 mg (356)** ** 300 mg (350)**	93.6%	**39.3%** **57.6%** 18.2 (11.1 to 25.4) **61.4%** 22.1 (14.9 to 29.2)

Chronic migraine is defined as > 15 monthly migraine days (MMD). Retention refers to participants completing the double-blind treatment phase. Efficacy is defined as the proportion of patients achieving a ≥ 50% reduction in MMD at the primary efficacy endpoint of each trial. Odds ratios (ORs) are reported relative to placebo with 95% confidence intervals.

**Table 3:** Pivotal clinical trials of gepants for the acute treatment of episodic migraine

Drug	Key clinical trial	*N*	Retention (%)	Pain freedom at 2hrs	Pain relief at 2hrs
**Ubrogepant** CGRP receptor Small-molecule CGRP receptor antagonist	**ACHIEVE-1** (2019) [134]	**Placebo (456)** ** 50 mg (423)** ** 100 mg (448)**	98.5%	**11.8%** **19.2%** **21.2%**	**49.1%** **60.7%** **61.4%**
**Rimegepant** CGRP receptor Small-molecule CGRP receptor antagonist	**Croop et al.,** (2019)[135]	**Placebo (682)** ** 75 mg (669)**	99.5%	**10.9%** **21.2%**	**59.3%** **43.3%**

Episodic migraine (EM) is defined as < 15 monthly migraine days (MMD). Retention refers to participants completing the double-blind treatment phase. Efficacy is defined as the proportion of patients achieving freedom from pain at 2 hours after treatment of a qualifying migraine attack. Primary end point efficacy is defined as the proportion of patients achieving freedom from pain at 2 hours after treatment of a qualifying migraine attack. Pain relief at 2 hours. Secondary end point pain relief at 2 hours (from moderate/severe to mild or no intensity).

**Table 4:** Pivotal clinical trials of gepants for the prophylaxis of migraine

Drug	Key clinical trial	*N*	Retention (%)	Primary endpoint (≥ 50% responder rate) OR vs placebo (95% CI) where reported
**Rimegepant** CGRP receptor Small-molecule CGRP receptor antagonist (oral)	**Croop et al.,** (2021) [136] 3 months Prior preventative use: NR Baseline MMD: 10.1/month (EM)	**Placebo (347)** ** 75 mg (348)**	84.5%	**41.0%** **49.0%**
**Atogepant** CGRP receptor Small-molecule CGRP receptor antagonist (oral)	**ADVANCE** (2021) [137] 3 months Prior preventative use: 70.3% Baseline MMD: 7.4/month (EM)	**Placebo (214)** ** 10 mg (214)** ** 30 mg (223)** ** 60 mg (222)**	88.5%	**29.0%** **55.6%** 3.1 (2.0 to 4.6) **58.7%** 3.5 (2.4 to 5.3) **60.8%** 3.8 (2.6 to 5.7)
	**PROGRESS** (2023) [138] 3 months Prior preventative use: 83% Baseline MMD: 16.0/month (CM)	**Placebo (246)** ** 30 mg twice a day (253)** ** 60 mg once a day (256)**	89%	**26%** **43%** 2.1 (1.5 to 3.1) **41%** 2.0 (1.4 to 3.0)

Episodic migraine (EM) is defined as < 15 monthly migraine days (MMD). Chronic migraine (CM) is defined as > 15 monthly migraine days (MMD). Retention refers to participants completing the double-blind treatment phase. Efficacy is defined as the proportion of patients achieving a ≥ 50% reduction in MMD at the primary efficacy endpoint of each trial. Odds ratio (ORs) are reported relative to placebo with 95% confidence intervals (CI). *NR* not reported

More pertinent are effects in more refractory groups. Efficacy has been observed in individuals with prior preventative treatment failures and in those with MOH, a group traditionally associated with poorer outcomes. In studies comparing MOH with no MOH, it is proposed that withdrawal is not necessary and treatment responses are comparable (Table 5). Yet MOH remains a risk factor for failure and relapse (Table 6), functional ability does not necessarily correspond, and a significant proportion remain non-responders.

**Table 5:** Phase III subgroup analyses of CGRP monoclonal antibodies in CM with medication overuse headache (MOH)

Drug Key Clinical Trial	*N*	Primary endpoint (≥ 50% reduction in MMDs) OR vs Placebo (95% CI) where reported	HIT-6 Difference from placebo (95% CI and *P* values where reported)	N	Primary Endpoint (≥ 50% reduction in MMDs) OR vs placebo (95% CI) where reported	HIT-6 Difference from placebo (95% CI and *P* values where reported)
**Erenumab** **Tepper et al.,** (2019) [139] 3 months	**MOH (**Baseline MMDs - 19.0)			**No MOH** (Baseline MMDs - 17.3)		
	**Placebo** (113) (54%) *	**17.7%**		**Placebo** (168) (47%) *	**27.4%**	
	**70mg** (77) (57%) *	**36.4%** 2.67 (1.36 to 5.22) *P* = 0.004	**−2.3** (−4.2 to −0.3) *P* = 0.021	**70mg** (111) (43%) *	**42.3%** 1.95 (1.17 to 3.23) *P* = 0.010	**−2.6** (−4.2 to −1.0) *P* = 0.002
	**140mg** (78) (51%) *	**34.6%** 2.51 (1.28 to 4.94) *P* = 0.007	**−2.5** (−4.4 to −0.6) *P* = 0.011	**140mg** (109) (46%) *	**45.9%** 2.25 (1.36 to 3.74) *P* = 0.002	**−2.5** (−4.1 to −0.8) *P* = 0.003
**Fremanezumab** **HALO** (2020) [140] 3 months	**MOH (**Baseline MMDs - 17.3)			**No MOH (**Baseline MMDs - 14.9)		
	**Placebo** (188) (20.2%) **	**13.8%**		**Placebo** (183) (21.3%) **	**22.4%**	
	**Quarterly** (201) (23.4%) **	**34.8%** 3.33 (2.01 to 5.52) *P* < 0.0001	**−1.5** *P* = 0.0356	**Quarterly** (174) (17.2%) **	**40.8%** 2.37 (1.50 to 3.76) *P* = 0.0003	**−2.4** *P* = 0.006
	**Monthly** (198) (27.3%) **	**39.4%** 4.08 (2.47 to 6.75) *P* < 0.0001	**−2.4** *P* = 0.0009	**Monthly** (177) (17.5%) **	**42.4%** 2.53 (1.60 to 4.00) *P* < 0.0001	**−2.3** *P* = 0.0012
**Galcanezumab** **REGAIN** (2021) [141] 3 months	**MOH (**Baseline MMDs - 20.0)			**No MOH (**Baseline MMDs - 18.3)		
	**Placebo** (353)	**13.8%**		**Placebo** (204)	**17.4%**	NR
	**120mg** (178)	**27.1%** *P* < 0.0001	NR	**120mg** (99)	**27.2%** *P* < 0.0001	
	**240mg** (177)	**27.4%** *P* < 0.0001		**240mg** (99)	**26.1%** *P* < 0.0001	

All studies are post hoc subgroups of placebo-controlled phase III randomised controlled trials. Participants met the ICHD-3 criteria for CM with medication overuse, had ≥ 15 headache days/month (including ≥ 8 migraine days) and had previous preventive treatment failure. Efficacy is defined as the percentage of patients achieving a ≥ 50% reduction in monthly migraine days (MMD). NR = not reported *% failure of ≥ 2 preventatives. **% current preventive use. HIT-6 : Headache Impact Test; meaningful response in CM −6-point reduction[142].

**Table 6:** Real-world effectiveness of CGRP monoclonal antibodies for migraine prevention

Study	Drug (*N*)	Migraine population	Baseline treatment history	Follow-Up	≥ 50% responder rate (MHD/MMD)	HIT-6 score
**EUREkA** Caronna et al., (2024)[143] (Europe) (35 Centres)	**Erenumab** **(2697)** **Fremanezumab (1486)** **Galcanezumab (1635)** N **=** 5818 inefficacy analysis	EM: 1620 (27.8%) CM: 4198 (72.2%) MOH: 3642 (62.6%)	Prior preventative failures median: 4 Previous BT-A: 60.8%	6 months	**56.5% (MHD)**	Baseline: **67.0** Endpoint: **NR**
	Lower baseline migraine frequency and disability were associated with a greater likelihood of ≥ 50% response					
**TRIUMPH** Lipton et al., (2026)[144] (International) (7 Countries)	**Galcanezumab (1105)** N = 1105 in efficacy analysis	EM: 332 (30.0%) CM: 773 (70.0%) MOH: NR	Prior preventative failures mean: 2.0	3 months	**EM: 51.8%** **(MMD)** **CM: 37.9%** **(MMD)**	**NR**
	Galcanezumab showed greater 3-month efficacy than traditional oral migraine preventives in routine clinical practice					
**GALCA-ONLY** Obach et al., (2023)[145] (Spain) (12 Centres)	**Galcanezumab (1055)** N = 1055 in efficacy analysis	HFEM: 249 (23.6%) CM: 806 (76.4%) MOH: NR	Prior preventative failures median: 4 Treatment-resistant migraine: 90.7%	12 months	**48.8% (MHD)** **HFEM: 58.2%** **CM: 45.9%**	**NR**
	Effectiveness was maintained in a highly treatment-resistant population including patients commonly excluded from clinical trials					
**FINESSE** Straube et al., (2025)[146] (Germany/Austria) (123 Centres)	**Fremanezumab (1016)** N = 987 in efficacy analysis	EM: 559 (55.0%) CM: 457 (45.0%) MOH: NR	Prior preventative therapy: 98.8% Previous BT-A: 38.4% Previous CGRP mAb: 16.5%	24 months	**52.8% (MMD)** **EM: 57.0%** **CM: 47.8%**	Baseline: **65.9** Endpoint: **55.8**
	Clinical response and improvements in headache-related disability were sustained over 24 months					
**PEARL** Ashina et al., (2025)[147] (Europe) (87 Centres)	**Fremanezumab (1140)** N = 968 in efficacy analysis	EM: 320 (33.1%) CM: 648 (66.9%) MOH: NR	Previous BT-A: 35.5% Previous CGRP mAb: 9.2%	12 months	**58.5% (MMD)** **EM: 67.7%** **CM: 54.1%**	Baseline: **66.8** Endpoint: **55.4**
	Approximately one-third of patients switching from another CGRP mAb achieved ≥50% MMD response					
**Barbanti et al., (2022)**[66] (Italy) (20 Centres)	**Erenumab** **(639)** **Fremanezumab** **(52)** **Galcanezumab (173)** N **= 864 in efficacy analysis**	HFEM: 208 (24.1%) CM: 656 (75.9%) MOH: 571 (87% of CM)	Prior preventative failures mean: 4.9	6 months	**HFEM: 64.9% (MMD)** **CM: 61.4%(MHD)**	Baseline: **66.0** Endpoint:**NR**
	Trigeminal sensitisation features predicted greater response whilst obesity predicted poorer response in CM					

Studies included prospective, multicentre real-world cohorts evaluating CGRP monoclonal antibodies in large patient populations. Responder rates represent a ≥ 50% reduction in monthly headache days (MHD) or monthly migraine days (MMD) as defined in the individual studies. Where available, outcomes are presented separately for episodic migraine (EM), high-frequency episodic migraine (HFEM) and chronic migraine (CM). HIT-6 scores are reported at baseline and follow-up where available. *BT-A* onabotulinumtoxinA, *CGRP* calcitonin gene-related peptide, *HIT-6* Headache Impact Test-6, *mAb* monoclonal antibody, *MOH* medication overuse headache, *NR* not reported

Gepants predated the mAbs but were directed back to the bench when abnormal liver function tests were seen with regular use [50]. Resurrection of the drug now offers oral acute and preventive treatment with Rimegepant and Atogepant (Table 3 and 4). Ubrogepant is effective acutely. Furthermore, the PRODROME trial suggested that Ubrogepant administered during the prodromal phase reduces progression to moderate or severe headache [51]. Compared to triptans, gepants seem better tolerated and lack vasoconstrictive properties, making them suitable for patients with vascular risk factors [52]. However, network meta-analyses indicate gepants are less effective than triptans in achieving pain freedom at 2 hours. Emerging clinical and preclinical evidence suggests gepants are unlikely to be associated with MOH and may reduce acute medication use although dedicated long-term data remain limited [53–56].

Despite the robust efficacy of CGRP-targeted therapies in RCTs, these findings may not fully translate to clinical practice. Trial populations are highly selected and contextual effects are estimated to account for up to 60% of the observed benefit [57]. Follow-up durations are relatively short, limiting conclusions about long-term durability, remission and disease modification. Patients treated in routine practice often present with more long-standing disease, higher baseline migraine frequency and MOH [58].

No CGRP mAb has been shown to be more effective than another, but direct comparisons are lacking. Network meta-analyses of phase III trials suggest numerically higher responder rates with fremanezumab [47, 59], but in CM, each mAb reduced MMD by 2 days more than placebo (range 1.8–2.3), with overlapping confidence intervals [60]. A real-world systematic review [61] also did not favour a particular agent. Furthermore, the first head-to-head study of a mAb and gepant, comparing galcanezumab with rimegepant in EM, demonstrated ≥ 50% responder rates in approximately 60% of patients, with no significant difference between the therapies [62](Table 7). Switching from a receptor (erenumab) to a ligand antibody (galcanezumab or fremanezumab) produced a ≥ 30% reduction in MMD in a third to a half of non-responders in small case series. None with daily headache responded [63, 64].

**Table 7:** Head-to-head randomised controlled trial comparing galcanezumab and rimegepant for the prophylaxis of episodic migraine

Study	Drug (*N*)	Retention (%)	Primary endpoint (≥ 50% responder rate) OR (95% CI) where reported	Key findings
**Schwedt et al.,** (2024) [62] 3 months (USA)	**Galcanezumab** (287) **Rimegepant (**293) Prior preventative use: 64% Baseline MMD: 8.4/month	**90.9%**	**62%** 1.1 (0.8 to 1.4) **61%**	• First head-to-head CGRP-targeted RCT • Comparable efficacy between galcanezumab and rimegepant

Participants fulfilled ICHD-3 criteria for episodic migraine (4–14 migraine days/month), were CGRP-targeted therapy naïve, and had not received onabotulinumtoxinA in the head or neck area within 3 months before enrolment. Episodic migraine (EM) is defined as <15 MMD per month. Retention refers to participants completing the double-blind treatment phase. *MMD* monthly migraine days

Conversely, 30–50% of EM patients and 40–60% with CM do not achieve a ≥ 50% response in routine practice (Table 6), and 15–25% discontinue because of lack of efficacy [61]. In a scoping review and meta-analysis of 38 real-world studies [65], daily headache (OR 0.24), depression (OR 0.51), other psychiatric comorbidities, and more failed preventive treatments predicted poorer response. Obesity in CM [66], interictal allodynia [67], higher baseline migraine frequency and medication overuse [68], smoking, hypertension and insomnia [69] have also been associated with poorer response, and unilateral pain with cranial autonomic symptoms with better response [66]. In EUREkA, those who stopped for the lack of effectiveness had more baseline headache days (median 25 vs. 20), more often had aura (23 vs. 16%), depression (38 vs. 23%) and obesity (19 vs. 7%) [70]. Additionally, pharmacogenomic analyses have identified single-nucleotide polymorphisms in genes involved in neuronal development that are associated with treatment [71].

One of the most pressing and open questions is whether CGRP-targeting therapies are disease-modifying or primarily suppress symptoms. In many centres, it has been common practice to pause treatment, typically after one year. Several studies [72–74] report that following discontinuation, the majority of patients relapse towards their baseline rates, suggesting a limited disease-modifying effect. However, the I-GRAINE study demonstrated that over three discontinuation periods, responder rates improved and migraine frequency decreased, supporting the possibility of cumulative benefits with continued use [75]. This carries a degree of optimism to the otherwise disheartening results for long-term disease modification in a lifelong disorder, with peak prevalence during working lives [76]. Thus, a significant proportion of individuals fail CGRP-directed treatments. It is this group that contributes disproportionately to the global burden of disease [1].

## Comorbidities, disease modification and a biopsychosocial approach to patient management

Unfulfilled expectations [77, 78] and failure of drug treatment often lead clinicians and patients to seek novel and often interventional approaches despite potential complications and disappointing results in RCTs [79]. The use of GONBs against placebo in CM shows self-limiting benefit acutely only [80]. Occipital neurostimulation studies in migraine [81] and cluster headache [82] have not shown benefits compared to sham. There is some evidence for external devices, mainly in acute treatment of migraine and cluster headache with some impact on prevention, such as peripheral vagal nerve stimulation [83]. Limitations include variable access, greater effect in episodic headache and use mainly in those most intractable.

The complexities of chronification and drug resistance in chronic pain have long been studied outside of neurology and recognised within the remit of the biopsychosocial (BPS) framework [84]. Although later in recognition, the BPS model is gaining traction in headache medicine [85, 86]. This model proposes a shift from a predominant biomedical approach to incorporate the social, psychological, and behavioural dimensions of illness [87]. Whilst a purely biomedical stance often assumes such factors ensue consequentially, there are BPS predispositions and ongoing risk factors, which underpin the route to chronification. What emerges is that such a framework is fundamental to all chronic pain disorders, which are comorbid and share risk factors.

Based upon the seminal work by Felitti [88], there is a cumulative risk of primary headaches in adulthood, episodic and chronic, in association with increasing adverse childhood events (ACEs) [89]. In one study of 1348 migraineurs, 34% had chronic daily headache, 26% evolving from episodic and 8% daily from onset. Physical and emotional ACEs were significantly associated with chronicity, emotional with continuous daily headache and greater headache-related disability. There was also a graded relationship with remote or ongoing anxiety, depression, obesity [90] and other primary pain syndromes [91]. The latter have been consistently reported to be comorbid with migraine and TTH. These include irritable bowel syndrome, fibromyalgia, chronic fatigue, temporomandibular disorders, low back and pelvic pain [92]. Furthermore, individuals with one primary headache are more likely to experience another primary headache [93, 94]. Complicating management is that frequent acute-relief medication use, irrelevant of what it is taken for, can exacerbate migraine and TTH in those predisposed [95]. A higher number of ACEs have been shown to be a poor prognostic indicator for ongoing MOH and headache chronicity. In those where there is no alteration in symptoms following withdrawal, poor prognostic factors include major depression and more severe stressful life events in the prior 10 years [96]. A proportion of those with MOH will fulfil criteria for substance dependency [97]. Focussed candidate gene association studies have shown polymorphic variants implicated in the dopaminergic system and overlap of genes associated with drug-dependence pathways [98].

A 2019 systematic review addressed modifiable risk factors for progression from episodic to CM or development of new-onset chronic daily headache (migraine ± TTH, purely TTH, and or hemicrania continua) [99]. The strongest risk factors were depression and acute-relief medication overuse. Moderate evidence implicated obesity, severity-graded asthma, snoring, non-cephalic pain and acute treatment efficacy. A 2025 systematic review confirmed similar risk factors for onset of migraine: premorbid atopia, sleep, cardiovascular and psychiatric disorders which included depression (bidirectional relationship), anxiety, autistic spectrum disorder and attention-deficit disorder (ADHD) [100]. Consistent with this are results of the GWAS studies in migraine showing strong links with vascular and the psychiatric disorders of depression and ADHD [101].

Maladaptive responses to pain can further perpetuate chronicity. Fixed pain beliefs, catastrophising and interference in daily activities correlate with perceived magnitude of pain [102]. Similarly, comparing CM to EM, psychosocial variables associated with greater bearing on disability include perceived headache impact, emotional distress, coping (catastrophising component), illness perceptions and the locus of control [103–105]. Such characteristics are also seen independent to psychiatric comorbidities [106].

It is the factors above which are prevalent in those patients failing treatment even with the newer CGRP-targeted treatments. Despite this, investment in non-pharmacological management is often overlooked and has little, if any, mention in clinical guidance [107].

A Cochrane review of psychological interventions in migraine concluded that it was not possible to determine whether psychological interventions are effective in managing migraine [108]. The same authors republished, citing the review to be limited by stringent inclusion criteria, and hence low power. They concluded psychological treatment may be of benefit in migraine and TTH, but better-quality studies were needed [109, 110]. It is unlikely that a single approach will address the complexities of human psychology, which is where a personalised approach is needed [111].

Independently, there is evidence for the addition of psychotherapy to usual care for depression, in terms of responses and remission [112]. Antidepressant treatment is less effective than psychological and non-pharmacological therapies in mild depression; in severe depression drug treatment alone, psychological and non-pharmacological therapies alone or a combination show an effect.

A meta-analysis of sleep in migraine demonstrated higher subjective sleep quality scores as indicated by the Pittsburgh sleep quality index. There were greater effects in CM, less rapid eye movement sleep as a percentage of total sleep time [113] and cutaneous allodynia, as a marker of sensitisation. Psychological sleep interventions have shown to improve headache frequency and sleep in migraine, TTH [114] and depression [115]. Pharmacological interventions can have short-term benefits in insomnia but are often poorly tolerated and there is limited information about longer-term outcomes [116]. When more formally assessed, there does not seem to be an increased risk of headache in obstructive sleep apnoea [117].

There is higher prevalence of migraine with obesity and being underweight [118] with greater cutaneous allodynia shown in those with obesity [119]. A meta-analysis of surgical and behavioural intervention showed both to impact on migraine frequency, pain severity, disability, and attack duration in adults and children. This was unrelated to baseline adiposity nor extent of weight loss [120].

Structural longitudinal imaging shows alterations related to level of activity of the disorder, reflective of ongoing clinical processes and plasticity [121]. It is therefore not surprising that unless the risk factors for progression to CM are addressed that the ongoing challenges to maintain allostasis will inevitably result in drug resistance. Delivery of a BPS model of management requires patient acceptance [122] and with engagement potentially sustained benefits in adults and children [123, 124]. With this model using multimodal approaches, we may be able to more comprehensively understand the complexities of CM [125].

### Summary

Migraine has a genetic predisposition. Although there are vascular associations, the evidence supports it to be a primarily central neurological disorder with altered nociceptive antinociceptive processing. Engineered disease-specific drugs have delivered the most effective and tolerated treatments, but these are not disease-modifying. There is significant contribution of psychosocial factors that may be responsible for the nociplasticity, which drives chronicity and refractoriness to drug treatment.
